# Supplementary material for: Correcting for Intra-Individual Variability in Sodium Excretion in Spot Urine Samples Does Not Improve the Ability to Predict 24 h Urinary Sodium Excretion
Source: Nutrients. 2020 Jul 8;12(7):2026. doi: 10.3390/nu12072026 (PMC7400094; doi:10.3390/nu12072026)
Supplement: Supplementary file 1 [file nutrients-12-02026-s001.pdf]

Supplementary Table S1. Spot potassium (K) and creatinine (Cr) urinary concentration distribution, raw data from 1 day, after adjustment for intra-individual variation of two and three spot sample collections and averages of multiple collections (N = 23)

	Raw Data for Day 1	Average of Days 1 and 2	Day 1 Corrected Using 2 Replicates	Average of Days 1, 2 and 3	Day 1 Corrected Using 3 Replicates
**Spot K (mmol/L)**					
Minimum	11.00	13.30	17.11	10.57	17.51
p5[a]	12.20	13.45	18.03	11.80	18.41
p10	12.70	13.75	18.40	13.57	18.77
p25	21.80	21.25	24.20	22.80	24.33
p50	26.70	32.60	26.82	28.93	26.82
p75	36.20	39.00	31.29	38.33	31.05
p90	50.40	51.65	37.00	50.57	36.40
p95	62.30	53.50	41.19	52.30	40.31
IQR	14.40	17.75	7.09	15.53	6.72
Maximum	65.30	62.50	42.18	64.97	41.23
Mean	30.47	32.31	27.82	31.50	27.73
SD	15.29	13.29	7.17	13.39	6.79
Spot Cr (mmol/L)					
Minimum	1.22	4.26	3.56	4.50	3.83
p5	3.31	4.74	5.39	4.65	5.58
p10	3.42	5.02	5.47	4.74	5.65
p25	4.06	6.63	5.87	6.86	6.02
p50	9.72	9.98	8.44	9.11	8.36
p75	12.58	12.33	9.40	11.67	9.21
p90	16.27	13.75	10.46	12.78	10.15
p95	19.38	14.15	11.25	17.75	10.84
IQR	8.52	5.70	3.53	4.81	3.19
Maximum	21.02	17.62	11.63	18.58	11.17
Mean	9.35	9.64	7.93	9.45	7.88
SD	5.47	3.61	2.13	3.80	1.93

[a] p =percentile. The sb/sobs was respectively for K and Cr: 0.507 and 0.416 when calculated using two replicates and 0.481 and 0.376 when calculated using three replicates.

Supplementary Table S2. Prediction equations to estimate 24hr Na, converted to salt equivalent (g/day) using correction applied for both spot Na and spot Cr using the INTERSALT equation

Statistics	Measured 24 h Na	INTERSALT Spot 1	INTERSALT Mean day 1 and 2	INTERSALT Adjusted day 1 and 2	INTERSALT Mean day 1, 2 and 3	INTERSALT Adjusted day 1, 2 and 3
Minimum	1.90	0.27	0.71	0.65	0.84	0.73
p5	2.90	1.25	2.31	2.51	2.87	2.69
p10	3.32	5.36	6.08	6.26	6.47	6.32
p25	3.91	7.87	7.06	7.25	7.15	7.24
p50	6.34	8.34	7.88	8.38	7.88	8.37
p75	9.12	9.93	9.76	9.77	10.21	9.83
p90	11.91	12.58	12.39	12.35	11.77	12.10
p95	13.03	12.78	12.39	12.66	12.30	12.21
IQR	5.21	2.06	2.69	2.52	3.05	2.59
Maximum	13.74	13.05	12.75	12.70	13.55	12.64
Mean	6.81	8.34	8.30	8.46	8.33	8.42
SD	3.34	3.04	2.90	2.85	2.83	2.78
p-value*		0.0208	0.0150	0.0150	0.0177	0.0163
Spearman r		0.3083	0.2856	0.2727	0.2885	0.2579
p-value		0.1524	0.1865	0.2080	0.1818	0.2348

* *p*-value assessed with Wilcoxon's signed rank test

Supplementary Table S3. Prediction equations to estimate 24hr Na, converted to salt equivalent (g/day), using correction applied for both spot Na and spot Cr using the Tanaka equation

Statistics	Measured 24 h Na	Tanaka Spot 1	Tanaka Mean day 1 and 2	Tanaka Adjusted day 1 and 2	Tanaka Mean day 1, 2 and 3	Tanaka Adjusted day 1, 2 and 3
Minimum	1.90	7.36	10.45	10.82	13.50	12.10
p5	2.90	12.28	13.38	13.69	14.51	14.00
p10	3.32	14.14	14.01	17.00	14.56	17.36
p25	3.91	16.89	16.80	19.26	16.38	19.71
p50	6.34	23.49	21.94	22.18	21.96	22.38
p75	9.12	28.60	24.59	25.99	25.54	24.88
p90	11.91	33.13	28.76	28.32	27.35	27.17
p95	13.03	33.92	29.62	29.50	28.29	28.36
IQR	5.21	11.72	7.79	6.73	9.16	5.17
Maximum	13.74	43.77	35.37	32.05	29.51	30.94
Mean	6.81	23.22	21.58	22.32	21.59	22.18
SD	3.34	8.23	5.77	5.02	4.96	4.43
p-value*		<0.0001	<0.0001	<0.0001	<0.0001	<0.0001
Spearman r		0.1047	0.3192	0.1808	0.2372	0.1927
p-value		0.6343	0.1377	0.4090	0.2759	0.3784

* *p*-value assessed with Wilcoxon's signed rank test

Supplementary Table S4. Prediction equations to estimate 24hr Na, converted to salt equivalent (g/day), using correction applied for both spot Na and spot Cr using the Kawasaki equation

**Statistics**	**Measured 24 h Na**	**Kawasaki Spot 1**	**Kawasaki Mean day 1 and 2**	**Kawasaki Adjusted day 1 and 2**	**Kawasaki Mean day 1, 2 and 3**	**Kawasaki Adjusted day 1, 2 and 3**
Minimum	1.90	7.70	12.04	12.59	16.69	14.51
p5	2.90	17.10	20.24	19.64	21.15	20.21
p10	3.32	20.69	22.10	23.91	21.74	25.05
p25	3.91	26.41	25.41	31.10	24.64	31.18
p50	6.34	40.31	36.45	40.62	36.78	39.77
p75	9.12	54.10	49.68	48.83	49.45	46.90
p90	11.91	61.20	55.64	53.72	58.67	53.55
p95	13.03	62.80	55.71	56.97	59.31	56.47
IQR	5.21	27.69	24.27	17.73	24.82	15.73
Maximum	13.74	80.55	57.77	65.32	60.05	62.13
Mean	6.81	41.07	37.54	39.10	37.65	38.79
SD	3.34	17.22	13.50	12.50	13.26	11.78
p-value*		<0.0001	<0.0001	<0.0001	<0.0001	<0.0001
Spearman r		0.1650	0.3192	0.2292	0.2806	0.2085
p-value		0.4518	0.1377	0.2927	0.1946	0.3397

* *p*-value assessed with Wilcoxon's signed rank test
